# Supplementary material for: Determinants of hypertension among patients with type 2 diabetes mellitus on follow-up at Tikur Anbessa Specialized Hospital, Addis Ababa: A case-control study
Source: PLoS One. 2021 Aug 23;16(8):e0256399. doi: 10.1371/journal.pone.0256399 (PMC8382175; doi:10.1371/journal.pone.0256399)
Supplement: S1 Questionnaire — (DOCX) [file pone.0256399.s002.docx]

***S1 Questionnaire***

1. ***English version***

Date of interview________ Participant’s Unique ID**_______**Name of data collector___________

| **Sr.No** | **Questions** | **Response and coding** | **Skip** |
| --- | --- | --- | --- |
|  | **Section I: Socio-demographic factors** |  |  |
| 101 | How old are you? | ______yrs |  |
| 102 | Sex | 1. Male 2. Female |  |
| 103 | Where is your residence? | 1. Urban 2. Rural |  |
| 104 | What is your current marital status? | 1. Single 2. Married 3. Divorced 4. Widowed |  |
| 105 | Which of the following best describes your **main work** status over the past 12 months? | 1. Government employee 2. Self-employed 3. House wife 4. Unemployed 5. Others (specify)……. |  |
| 106 | What is your level of education? | 1. No formal education 2. Primary school 3. Secondary school 4. Higher education |  |
| 107 | What is your average household monthly income? | _______________(Birr) |  |
| **Section II: Clinical factors** | | | |
| 201 | Date of DM diagnosis | Dd____mm_____yyyy______ |  |
| 202 | For how long have you been diabetic? | ______yr |  |
| 203 | Indicate the date of start for only those medications you ever used out of the following antidiabetic medications? | 1. Metformin__________ 2. Glibenclamide________ 3. Insulin____________ 4. Other, ___________ 5. Specify other_________ |  |
| 204 | How long have you been using the drugs you described above? Please Indicate the date you stopped taking the medicine for each of the followings that is applicable to you. | - 1. Metformin_________   2. Glibenclamide________   3. Insulin____________   4. Other ___________   5. Specify other_________ |  |
| 205 | How frequently are you ordered by your doctor to take anti-diabetic drug per day? | ________times/day |  |
| 206 | Have you ever missed any of the doses that was ordered by your doctor? | 1. Yes____ 2. No___ | 208 |
| 207 | If yes to question 204, how many doses have you missed in the last month? | _____________doses/month |  |
| 208 | Were there anytime that you have quitted your anti-diabetic medication? | 1. Yes____ 2. No___ | 210 |
| 209 | If yes to question no. 206, for how long have you quitted? | _______week |  |
| 210 | Are you on regular follow up for diabetes? | 0. Yes____  1. No___ |  |
| 211 | Do you attend diabetic education session? | 0. Yes____  1. No___ |  |
| 212 | Are you a member of diabetes association? | 0. Yes____  1. No___ |  |
| 213 | Do you have glucometer at home? | 0. Yes____  1. No___ |  |
| 214 | On how many of the last SEVEN DAYS did you test your blood sugar? | 0 1 2 3 4 5 6 7 |  |
| 215 | Do you have family history of hypertension? | - 1. Yes____   2. No___   3. I don’t know |  |
| 216 | Have you ever seen a traditional healer for diabetes or raised blood sugar? | 1. Yes____ 2. No___ | 218 |
| 217 | If yes to question no. 209, Are you currently taking any herbal or traditional remedy for your diabetes? | 1. Yes____  2. No___ |  |
| 218 | Do you have comorbidity confirmed by physician other than hypertension? | 1. Yes____  2. No___ |  |
| 219 | If yes to 216, what is the diagnosis?  (RECORD ALL with date of diagnosis) |  |  |
| **Section III: Life style and behavioral factors**  Now I am going to ask you some questions about various health behaviors. This includes things like smoking, drinking alcohol, diet and physical activity. Let's start with tobacco. | | | |
| 301 | Have you ever smoked any tobacco products, such as cigarettes, cigars or pipes? | 1. Yes____  0. No___ go to 307 |  |
| 302 | Do you currently smoke tobacco products daily? | 1. Yes____  0. No___ |  |
| 303 | How old were you when you first started smoking daily? DON’T KNOW 77 | _____years. If known, go to 305 |  |
| 304 | Do you remember how long ago it was? (RECORD ONLY 1, NOT ALL 3) DON’T KNOW 77 | ______yrs OR _____months OR____wks |  |
| 305 | On average, how many of the following do you smoke each day?  RECORD FOR EACH TYPE | Manufactured cigarettes_____  Hand-rolled cigarettes_____  Pipes full of tobacco________  Cigars, cheroots, cigarillos______  Other________  Please specify other_________ |  |
| 306 | How old were you when you stopped (if any) smoking daily? | _____years.  Don’t know, how long ago it was____ |  |
| **The next questions ask about the consumption of ALCOHOL.** | | | |
| 307 | Have you ever consumed an alcoholic drink such as beer, wine, spirits, fermented cider or tejj, tella, areke? | 1. Yes____  0. No___ go to 312 |  |
| 308 | During the past 12 months, how frequently have you had at least one alcoholic drink? | 1. Daily 2. 5-6 days per week 3. 1-4 days per week 4. 1-3 days per month 5. Less than once a month 6. Never |  |
| 309 | When you drank alcohol, on average, how many standard alcoholic drinks did you have during one drinking occasion? | ________drinks |  |
| 310 | What was the largest number of standard alcoholic drinks you had on a single occasion, counting all types of alcoholic drinks together? | Largest no. _________  Don't Know 77 |  |
| 311 | In a typical month, how many times did you have **for men: five or more**  **for women: four or more** standard alcoholic drinks in a single drinking occasion? | _________ times  Don't Know 77 |  |
| **DIET**: The next questions ask about diet that you usually eat. As you answer these questions, please think of a typical week in the last year. | | | |
| 312 | In a typical week, on how many days do you **eat fruit**? | _________days/wk |  |
| 313 | How many **servings** of fruit do you eat on **one** of those days? | ________ servings/day |  |
| 314 | In a typical week, on how many days do you eat vegetables? | _________days/wk |  |
| 315 | How many servings of vegetables do you eat on one of those days? | ________ servings/day |  |
| 316 | What type of oil or fat is most often used for meal preparation in your household? | 1. Vegetable oil 2. Cholestrol oil 3. Butter or ghee 4. Other specify……… |  |
| 317 | On average, how many meals per week do you eat that were not prepared at a home? By meal, I mean breakfast, lunch and dinner. | _______meals /wk |  |
| 318 | How is your salt consumption after DM diagnosis? | 1. No change from prediabetic period 2. Minimally decreased 3. Substantially decreased 4. Stopped at all |  |
| **Physical Activity:** Next I am going to ask you about the time you spend doing different types of physical activity in a typical week. Please answer these questions even if you do not consider yourself to be a physically active person. Think first about the time you spend doing work. Think of work as the things that you have to do such as paid or unpaid work, study/training, household chores, harvesting food/crops, fishing or hunting for food, seeking employment. | | | |
| 319 | Does your work involve vigorous-intensity activity that causes large increases in breathing or heart rate like [carrying or lifting heavy loads, digging or construction work] for at least 10 minutes continuously? | - - 1. Yes____     2. No___ | 322 |
| 320 | In a typical week, on how many days do you do vigorous-intensity activities as part of your work? | _______days |  |
| 321 | How much time do you spend doing vigorous-intensity activities at work on a typical day? | ____:____Hr:min /day |  |
| 322 | Does your work involve moderate-intensity activity that causes small increases in breathing or heart rate such as brisk walking [or carrying light loads] for at least 10 minutes continuously? | 0. Yes____  1. No___ | 325 |
| 323 | In a typical week, on how many days do you do moderate-intensity activities as part of your work? | ______days |  |
| 324 | How much time do you spend doing moderate-intensity activities at work on a typical day? | ____:_____Hr:min /day |  |
| The next questions exclude the physical activities at work that you have already mentioned. Now I would like to ask you about the usual way you **travel to and from places**. For example to work, for shopping, to market, to place of worship, to taxi station | | | |
| 325 | Do you walk or use a bicycle (pedal cycle) for at least 10 minutes continuously to get to and from places? | 0. Yes____  1. No___ | 328 |
| 326 | In a typical week, on how many days do you walk or use a pedal cycle for at least 10 minutes continuously to get to and from places?? | _________days |  |
| 327 | How much time do you spend walking or bicycling for travel on a typical day? | ____:_____Hr:min /day |  |
| The next questions exclude the work and transport activities that you have already mentioned. Now I would like to ask you about sports, fitness and **recreational activities** (leisure) | | | |
| 328 | Do you do any vigorous-intensity sports, fitness or recreational (leisure) activities that cause large increases in breathing or heart rate like [running or football or lifting weight] for at least 10 minutes continuously? | 0. Yes____  1. No___ | 331 |
| 329 | In a typical week, on how many days do you do vigorous-intensity sports, fitness or recreational (leisure) activities? | _____________days |  |
| 330 | How much time do you spend doing vigorous-intensity sports, fitness or recreational activities on a typical day? | ____:_____Hr:min /day |  |
| 331 | Do you do any moderate-intensity sports, fitness or recreational (leisure) activities that cause a small increase in breathing or heart rate such as brisk walking, [cycling, swimming, volleyball] for at least 10 minutes continuously? | 0. Yes____  1. No___ | 334 |
| 332 | In a typical week, on how many days do you do moderate-intensity sports, fitness or recreational (leisure) activities? | _____________days |  |
| 333 | How much time do you spend doing moderate-intensity sports, fitness or recreational (leisure) activities on a typical day? | ____:_____Hr:min |  |
| The following question is about sitting or reclining at work, at home, getting to and from places, or with friends including time spent sitting at a desk, sitting with friends, traveling in car, bus, train, reading, playing cards or watching television, but do not include time spent sleeping. | | | |
| 334 | How much time do you usually spend sitting or reclining on a typical day? (Sedentary behavior) | ____:_____Hr:min |  |
| 335 | How many hours of actual sleep do you get in a 24-hour period? | _________hr |  |
| **PERCEIVED STRESS SCALE፡** The following questions will ask you about your feelings and thoughts in the last month.  0 = Never 1 = Almost Never 2 = Sometimes 3 = Fairly Often 4 = Very Often | | | |
| No. | Questions | Score |  |
| 336 | In the last month, how often have you been upset because of something that happened unexpectedly? | 0 1 2 3 4 |  |
| 337 | In the last month, how often have you felt that you were unable to control the important things in your life? | 0 1 2 3 4 |  |
| 338 | In the last month, how often have you felt nervous and “stressed”? | 0 1 2 3 4 |  |
| 339 | In the last month, how often have you felt confident about your ability to handle your personal problems? | 0 1 2 3 4 |  |
| 341 | In the last month, how often have you felt that things were going your way? | 0 1 2 3 4 |  |
| 342 | In the last month, how often have you found that you could not cope with all the things that you had to do? | 0 1 2 3 4 |  |
| 343 | In the last month, how often have you been able to control irritations in your life? | 0 1 2 3 4 |  |
| 344 | In the last month, how often have you felt that you were on top of things? | 0 1 2 3 4 |  |
| 345 | In the last month, how often have you been angered because of things that were outside of your control? | 0 1 2 3 4 |  |
| 346 | In the last month, how often have you felt difficulties were piling up so high that you could not overcome them? | 0 1 2 3 4 |  |
| **Section IV: Measurements** | | | |
| 401 | Weight | _________kg |  |
| 402 | Height | _________cm |  |
| 403 | Waist circumference | _________cm |  |
| 404 | Hip circumference | _________cm |  |
| 405 | Serum triglyceride | _________mg/dl |  |
| 406 | Serum cholesterol | _________mg/dl |  |
| 407 | Serum HDL | _________mg/dl |  |
| 408 | Serum LDL | _________mg/dl |  |
| 409 | FBS 1______ FBS 2_____ FBS 3____ FBS4_____ | _________mg/dl |  |
| 410 | HA1C |  |  |
| 411 | Serum creatinine | _________mg/dl |  |
| 412 | BP from record | Systolic _________ ( mmHg)  Diastolic_________ (mmHg) |  |
| 413 | Reading 1 | Systolic_________mmHg)  Diastolic_________ (mmHg) |  |
| 414 | Are you taking drug for hypertension? | - - 1. Yes  1. No |  |
| 415 | Status | 1. Control 🡪 Thank you 2. Case 🡪416 |  |
| 416 | For **cases only**, How long have you been since diagnosed with hypertension? | ______year |  |

- ***Indicates information to be extracted from record***

**B. የአማርኛ መጠይቅ**

የተጠየቀው ቀን________የጥናቱ ተሳታፊ ኮድ______መረጃውን የሰበሰበሰው ስም________________

| **ተ.ቁ** | **ጥያቄዎች** | **መልስ እና ምስጢር ቁጥር** | **ዝለል** |
| --- | --- | --- | --- |
| **100** | **ክፍል I: አጠቃላይ መረጃ** |  |  |
| 101 | ዕድሜዎ ስንት ነው? | ______ዓመት |  |
| 102 | ጾታ (በማየት ይመዝግቡ) | 1. ወንድ 2. ሴት |  |
| 103 | መኖሪያዎ የት ነው? | 1. ከተማ 2. ገጠር |  |
| 104 | የአሁኑ የጋብቻ ሁኔታዎ ምንድነው? | 1. ያላገባ/ች 2. ያገባ/ች 3. የተፋታ/ች 4. ባል/ሚስት የሞተበት |  |
| 105 | የሥራዎ ሁኔታ ባለፉት 12 ወራት ምንድን ነበር? | 1. የመንግስት ሠራተኛ 2. የግል ሥራ 3. የቤት እመቤት 4. ሥራ-አጥ 5. ሌላ (ጥቀስ)________ |  |
| 106 | የትምህርት ደረጃዎት ምንድን ነው? | 1. አልተማርኩም 2. የመጀመርያ ደረጃ 3. ሁለተኛ ደረጃ 4. ከፍተኛ ትምህርት |  |
| 107 | የቤታችሁ አማካይ የወር ገቢ ምን ያህል ነው? | _____ ብር በወር |  |
| **ክፍል II፡ ከጤና ጋር የተያያዙ መረጃዎች** | | | |
| **201** | የስኳር በሽታ እነዳለብዎት ያወቁበት ቀን | ቀን____ወር_____ዓ/ም______ ከታወቀ ወደ ቁ203 |  |
| 202 | የስኳር በሽታ እንዳለብዎት ካወቁ ወዲህ ምን ያክል ጊዜ ሆነ? | _____ዓመት |  |
| 203 | ከሚከተሉት የስኳር መድኃኒቶች ተጠቅመው ለሚያውቁት ብቻ የጀመሩበትን ቀን ይግለጹ? | 1. ሜትፎርሚን______ 2. ዳኦኒል/ግሌብንክላማይድ­­­­______ 3. እንሱሊን______ 4. ሌላ____________ |  |
| 204 | ከላይ እንደጀመሩ የገለጹትን መድኃኒቶች እስከመቼ ድረስ ተጠቀሙ (እየተጠቀሙ ነው)? (እባክዎን እርስዎን ለሚመለከት ለእያንዳንዱ ተከታይ መድሃኒት መውሰድ ያቆሙበትን ቀን ያመልክቱ) | 1. ሜትፎርሚን______ 2. ግሌብንክላማይድ­­­­______ 3. እንሱሊን______ 4. ሌላ____________ |  |
| 205 | ዶክተርዎ የስኳር መድሀኒትዎን በቀን ስንቴ እንዲወስዱ አዘዝዎት? | ____________ ጊዜ በቀን |  |
| 206 | ከታዘዙት መጠን ማንኛውንም ጊዜ ቢሆን ሳይወስዱ ቀርተው ያውቃሉ ? | 1. አዎን____  0. አላውቅም___ |  |
| 207 | ለቁጥር 206 መልስዎ አዎ ከሆነ ባለፈው ወር ሳይወስዱ ያለፈብዎት የስንት ጊዜ ይሆናል? (ሁሉንም ይቁጠሩ) | ____________ ጊዜ በወር |  |
| 208 | የስኳር በሽታ መድሐኒት አቋርጠው ያውቃሉ ? | 1. አዎን____ 2. አላውቅም___ | 210 |
| 209 | ለቁጥር 208 መልስዎ አዎ ከሆነ ለምን ያህል ግዜ? | ለ____ ሳምንት |  |
| 210 | ለስኳር በሽታ መደበኛ ክትትል ላይ ኖት? | 1. አዎን____ 2. አይደለሁም___ |  |
| 211 | በስኳር በሽታ ትምህርት ክፍለ ጊዜ ላይ ይሳተፋሉ? | 1. አዎን 1. አልሳተፍም |  |
| 212 | የስኳር ህመም ማህበር አባል ነዎት? | 1. አዎን 1. አይደለሁም___ |  |
| 213 | የስኳር መጠን መለክያ በቤትዎ አለዎት? | 1. አዎን 1. አይደለም |  |
| 214 | ባለፈው ሰባት ቀናት ውስጥ የስኳርዎን መጠን ስንቴ ለኩ? | 0 1 2 3 4 5 6 7 |  |
| 215 | በቤተሰብዎ ታሪክ የደም ግፊት ያለበት ሰው አለ? | 1. አዎን 2. የለም 3. አላውቅም |  |
| 216 | ለስኳር በሽታዎ የባህል ሃኪም ጋር ሄደው ያዉቃሉ? | 1. አዎን 2. አይደለም | 218 |
| 217 | ለቁጥር 216 መልስዎ አዎ ከሆነ አሁን ለስኳር በሽታዎ የባህል ወይም የልምድ መድሃኒት ይወስዳሉ? | 1. አዎን 2. አይደለም |  |
| 218 | ከደም ግፊት ሌላ በሀኪም የተረጋገጠ ተደራቢ በሽታ አለብዎት? | 1. አዎን 2. የለም___ |  |
| 219 | ለቁጥር 218 መልስዎ አዎ ከሆነ የምርመራው ውጤት ምን ነበር? ( የምርመራ ውጤት እና የተመረመሩበት ቀን ይመዝገብ) |  |  |
| **300** | **ክፍል III: የህይወት ዘይቤ እና የባህሪ ምክንያቶች**  አሁን ስለ የተለያዩ የጤና ጠባይ አንዳንድ ጥያቄዎችን ልጠይቅዎት እፈልጋለሁ ፡፡ ይህ እንደ ማጨስ ፣ አልኮልን መጠጣት ፣ አመጋገብ እና የአካል እንቅስቃሴ ያሉ ነገሮችን ያጠቃልላል። ከትንባሆ እንጀምር ፡፡ | | |
| 301 | እንደ ሲጋራና የመሳሰሉ ማናቸውንም ዓይነት የትምባሆ ምርቶች አጭሰው ያውቃሉ? | 1. አዎን 2. አይደለም- (ወደ 307 ይሂዱ) |  |
| 302 | በአሁኑ ጊዜ የትምባሆ ምርቶችን በየቀኑ ያጨሳሉ? | 1. አዎን   1. አይደለም |  |
| 303 | በየቀኑ ማጨስ ሲጀምሩ ዕድሜዎ ስንት ነበር? | _____ዓመት፡፡ ምታወቅ ከሆነ ወደ 305 |  |
| 304 | ማጨስ የጀመርከው ከምን ያክል ጊዜ በፊት እንደነበር ታስታውሳለህ? | ____________ |  |
| 305 | በአማካይ በየቀኑ ከሚከተሉት ውስጥ ምን ያህል ያጨሳሉ?  የትኞችን የትንባሆ ዓይነት ከሆነ (ከአንድ በላይ መመለስ ይቻላል) | በፋብሪካ የተመረቱ ሲጋራዎች_  በእጅ የተጠቀለሉ ሲጋራዎች___  ጋያ________  ሌላ________  እባክዎ ሌላ ከሆነ ስሙን ይጥቀሱ_______ |  |
| 306 | ማጨስ አቁመው ከሆነ ያቆሙት በስንት ዓመትዎ ነበር?  ማጨስ ስያቆሙ ዕድሜዎ ስንት እንደነበረ ካላወቁ ማጨስ ካቆሙ ምን ያክል ጊዜ ሆነው____ | _____ዓመት  ካላወቁ ምን ያክል ጊዜ ሆነው____ |  |
| **አልኮሆል:** የሚቀጥሉት ጥያቄዎች ስለ አልኮሆል ፍጆታ ይጠይቃሉ ፡፡ | | | |
| 307 | የአልኮል መጠጥ ጠጥተው ያውቃሉ? ( ቢራ ፣ ወይን ፣ ጠላ፣ጠጅ፣ አረቄ…) | 1. አዎን  0. አይደለም | 312 |
| 308 | በአለፉት 12 ወራት ውስጥ ቢያንስ አንድ የአልኮል መጠጥ የሚጠጡት ስንት ጊዜ ነበር? | 1. በየቀኑ 2. 5-6 ቀናት በሳምንት 3. 1-4 ቀናት በሳምንት 4. 1-3 ቀናት በወር 5. በወር ከአንዴ በታች 6. ጠጥቼ አላውቅም |  |
| 309 | የአልኮል መጠጥ በሚጠጡበት ጊዜ በአማካይ ስንት አልኮሆል መጠጦች በአንድ የመጠጥ ጊዜ ውስጥ ይጠጣሉ? | _________ |  |
| 310 | በአንድ የመጠጥ ጊዜ ትልቁ የጠጣሃቸው የአልኮል መጠጦች ሁሉንም የአልኮል መጠጦችን በመቁጠር ስንት ነበሩ? | ትልቁ የጠጣሁት__________ |  |
| 311 | በተለመደ ወር ውስጥ **ወንድ 5እና ከዚያ በላይ**  ***ሴት 4እና ከዚያ በላይ*** የአልኮሆል መጠጥ በአንድ የመጠጥ ጊዜ የወሰዱት ምን ያህል ጊዜ ነው? |  |  |
| **አመጋገብ፡** የሚቀጥሉ ጥያቄዎች አብዛኛውን ጊዜ ስለሚመገቡት የፍራፍሬና የአትክትልት አመጋገብ ይጠይቅዎታል፡፡ እባክዎን መልስዎን ሲመልሱ ባለፈው ዓመት ውስጥ የእርስዎን አመጋገብ ስለሚወክለው ሳምንት ያስቡ፡፡ | | | |
| 312 | በተለመደው ሳምንት ምን ያክል ቀን ፍራፍሬ ትመገባለህ? (እባክዎን ካርድ ያሳዩ) | _________ቀን |  |
| 313 | ከእነኚህ ቀናቶች በአንዱ ላይ ምን ያክል የፍራፍሬ ገበታ ይመገባሉ? (እባክዎን ካርድ ያሳዩ) | _________ |  |
| 314 | በተለመደው ሳምንት ምን ያክል ቀን አትክልት ትመገባለህ? (እባክዎን ካርድ ያሳዩ) | _________ቀን |  |
| 315 | ከእነኚህ ቀናቶች አንዱ ላይ ምን ያክል የአትክልት ገበታ ይመገባሉ? (እባክዎን ካርድ ያሳዩ) | _________ |  |
| 316 | ብዙ ጊዜ ምግብ ለማብሰል ምን አይነት ዘይት ነው የምትጠቀሙት? | 1. የአትክልት ዘይት 2. የምረጋ ዘይት 3. ቂቤ 4. ሌላ……………. |  |
| 317 | በሳምንት ከቤት ውጪ በአማካይ ምን ያክል ጊዜ ይመገባሉ? ቁርስ፣ምሳና እራት ተቆጥረው ተቆጥረው ማለቴ ነው | ________ጊዜ በሳምንት |  |
| 318 | የስኳር በሽታ እንዳለብዎት ከተነገርዎት በኋላ የጨው ፍጆታዎ እንዴት ነው? | 1. በፊት ከሚጠቀሙት መጠን ለውጥ የለውም 2. በጥቂቱ ቀንሰዋል 3. በጣም ቀንሰዋል 4. መጠቀም አቁመዋል |  |
| **የአካል ብቃት እንቅስቃሴ-** ቀጣይ በተለመደ ሳምንት ውስጥ የተለያዩ የአካል ብቃት እንቅስቃሴ ዓይነቶችን በመሥራት ያሳለፉትን ጊዜ እጠይቅዎታለሁ ፡፡ ምንም እንኳን እርስዎ አካላዊ እንቅስቃሴ የሚያደርጉ ሰው ባይሆኑም እባክዎን እነዚህን ጥያቄዎች ይመልሱ ፡፡ ሥራን ስለሚሰሩበት ጊዜ በመጀመሪያ ያስቡ ፡፡ እንደ ደመወዝ ወይም ያልተከፈለ ሥራ ፣ ጥናት / ስልጠና ፣ የቤት ውስጥ ሥራዎች ፣ ምግብ / ሰብሎች መሰብሰብ ፣ ዓሳ ማጥመድ ወይም ምግብ መፈለግ ላይ እንደመሰማራት ያሉ መሥራት ያለብዎት ነገሮችን ያስቡ ፡፡ | | | |
| 319 | ሥራዎ አተነፋፈስዎን ወይንም ልብ ምትዎን በጣም ሊጨምር የሚችል ከባድ እንቅስቃሴን ለምሳሌ እንደመሸከም ከባድ ነገርን እንደማንሳት ወይንም እንደ ግንባታ ሥራ ቢያንስ ለ10 ደቂቃ ያለማቋረጥ መስራትን ያካትታል? | 1. አዎን ____ 2. አይደለም___ | 322 |
| 320 | እንዲህ አይነት ከባድ ስራዎችን በሳምንት ምን ያክል ቀን ይሰራሉ? | ____ቀን |  |
| 321 | በሥራዎ ውስጥ በአንድ በተለመደ ቀን ከባድ እንቅስቃሴዎችን በመከወን ምን ያህል ጊዜ ያሳልፋሉ? | _____:______  (እባክዎን በደቂቃ ቀይረው ይመዝግቡ) |  |
| 322 | ሥራዎ የመተንፈስ ወይም የልብ ምት በትንሹ የሚጨሚሩ መካከለኛ እንቅስቃሴን እንደ [ፈጣን እርምጃ ወይም ቀላል ጭነቶችን እንደመሸከም] ያሉ እንቅስቃሴን ያለማቋረጥ ቢያንስ ለ 10 ደቂቃዎች መሥራትን ያካትታል? | 1. አዎን  2. አይደለም | 325 |
| 323 | በተለመደው ሳምንት ውስጥ እንደስራዎ አንድ ክፍል እንዲህ አይነት መካከለኛ-እንቅስቃሴዎችን ስንት ቀናት ያደርጋሉ? | ____ቀን |  |
| 324 | በሥራዎ ውስጥ በአንድ በተለመደ ቀን መካከለኛ እንቅስቃሴዎችን በመከወን ምን ያህል ጊዜ ያሳልፋሉ? | _____:______ |  |
| የሚቀጥሉት ጥያቄዎች ቀደም ሲል እርስዎ የገለፁትን በስራ ላይ ያሉትን የአካል ብቃት እንቅስቃሴዎችን አያካትቱም ፡፡ አሁን ወደ **ቦታዎች ለመሄድና እና ከቦታዎች ለመመለስ** ስለሚጓዙበት የተለመደ ጉዞ ልጠይቅዎ እፈልጋለሁ ፡፡ ለምሳሌ ለመስራት ፣ ዕቃ ለመግዛት ፣ ለገበያ ፣ ለአምልኮ ቦታ ፣ ለታክሲ መያዣ…ወዘተ | | | |
| 325 | ወደ ቦታዎች ለመሄድ እና ለመመለስ ብስክሌትን ወይንም የእግር ጉዞን ያለማቋረጥ ቢያንስ ለ 10 ደቂቃዎች ይጠቀማሉ? | 1. አዎን  2. አይደለም | 328 |
| 326 | በተለመደው ሳምንት ወደ ቦታዎች ለመሄድ እና ለመመለስ ብስክሌትን ወይንም የእግር ጉዞን ያለማቋረጥ ቢያንስ ለ 10 ደቂቃዎች ስንት ቀን ይጠቀማሉ? | ____ቀን |  |
| 327 | በተለመደው ቀን ለጉዞ ብስክሌትን በመንዳት ወይንም የእግር ጉዞን በመጓዝ ምን ያህል ጊዜ ያሳልፋሉ? | _____:______ |  |
| የሚቀጥሉት ጥያቄዎች እርስዎ ቀደም ሲል የጠቀሷቸውን የሥራ እና የትራንስፖርት እንቅስቃሴዎችን አያካትቱም ፡፡ አሁን ስለ ስፖርት ፣ የአካል ብቃት እና **የመዝናኛ እንቅስቃሴዎች (መዝናኛ)** ልጠይቅዎት እፈልጋለሁ ፡፡ | | | |
| 328 | ቢያንስ ለ 10 ደቂቃዎች ያለማቋረጥ እንደ ሩጫ ወይም እግር ኳስ ወይም ክብደት ማንሳት ያሉ አተነፋፈስ ወይም የልብ ምት በከፍተኛ የሚጨምሩ ጠንካራ የስፖርት ፣ የአካል ብቃት ወይም የመዝናኛ እንቅስቃሴዎችን ያደርጋሉ? | 1. አዎን  2. አይደለም | 331 |
| 329 | በተለመደው ሳምንት ውስጥ ጠንካራ ስፖርት ፣ የአካል ብቃት ወይም የመዝናኛ እንቅስቃሴዎችን ስንት ቀናት ያደርጋሉ? | ____ቀን |  |
| 330 | በተለመደው ቀን ጠንካራ የስፖርት ፣ የአካል ብቃት እንቅስቃሴ ወይም የመዝናኛ እንቅስቃሴዎችን በማድረግ ምን ያህል ጊዜ ያሳልፋሉ? | _____:______ |  |
| 331 | ቢያንስ ለ 10 ደቂቃዎች ያለማቋረጥ እንደ ብስክሌት መንዳት ፣ መዋኘት ፣ የእጅ (የመረብ) ኳስ ያሉ አተነፋፈስ ወይም የልብ ምት በትንሹ የሚጨምሩ መጠነኛ የስፖርት ፣ የአካል ብቃት ወይም የመዝናኛ እንቅስቃሴዎችን ያደርጋሉ? | 1. አዎን  2. አይደለም | 334 |
| 332 | በተለመደ ሳምንት ፣ መጠነኛ ስፖርቶችን ፣ የአካል ብቃት እንቅስቃሴን ወይም የመዝናኛ እንቅስቃሴዎችን ስንት ቀናት ያደርጋሉ? | ____ቀን |  |
| 333 | በተለመደ ቀን መጠነኛ-ስፖርቶችን ፣ የአካል ብቃት እንቅስቃሴን ወይም የመዝናኛ እንቅስቃሴዎችን በማከናወን ምን ያህል ጊዜ ያሳልፋሉ? | _____:______ |  |
| የሚከተለው ጥያቄ በስራ ላይ ሆነው፣ በቤት ውስጥ፣ ከጓደኞች ጋር ፣ መኪና ውስጥ በመጓዝ ፣ በአውቶቡስ ፣ በባቡር ፣ በማንበብ ፣ ካርዶችን በመጫወት ወይም ቴሌቪዥን በመመለከት ተቀምጠው ስለሚያሳልፉ ጊዜ ነው ፡፡ ነገር ግን የመተኛት ጊዜን አይጨምርም። | | | |
| 334 | በተለመደው ቀን ለመቀመጥ ወይም ለማረፍ ምን ያህል ጊዜ ያሳልፋሉ? | ____: _____ ሰዓት: ደቂቃ |  |
| 335 | በ 24 ሰዓታት ውስጥ በአማካይ ምን ያህል ሰዓት ትክክለኛ እንቅልፍ ያገኛሉ? | ____: _____ ሰዓት: ደቂቃ |  |
| **የተገነዘቡት ወይንም የተስተዋለ የውጥረት ልክ፡** የሚቀጥሉ ጥያቄዎች ባለፈው ወር ውስጥ ስለተማዎት ስሜቶችዎ እና ሀሳቦችዎ ይጠይቅዎታል **፡፡**  **ውጤት 0=በጭራሽ 1=በጣም ጥቂት ጊዜ 2=አንድአንዴ 3=አብዛኛውን ጊዜ 4=ዘውትር** | | | |
| 1 | በሕይወትዎ ያልተጠበቀ ነገር በመከሰቱ ባለፈው ወር ውስጥ ምን ያህል ጊዜ ተበሳጭተዋል? | 0 1 2 3 4 |  |
| 2 | በሕይወትዎ ውስጥ አስፈላጊ የሆኑትን ነገሮች መቆጣጠር እንዳልቻሉ ባለፈውወር ውስጥ ምን ያህል ጊዜ ተሰምቶት ያውቃል? | 0 1 2 3 4 |  |
| 3 | ባለፈው ወር ውስጥ የመጨነቅ እና የመረበሽ ስሜት ምን ያህል ጊዜ ተሰምቶት ያውቃል? | 0 1 2 3 4 |  |
| 4 | ባለፈው ወር ውስጥ የግል ችግሮችዎን በራስዎ አቅም ለመፍታት ምን ያህል ጊዜ በራስ የመተማመን ስሜት ተሰምቶልዎት ያውቃል? | 0 1 2 3 4 |  |
| 5 | ባለፈው ወር ውስጥ ነገሮች እርስዎ በሚፈልጉት መንገድ እየሄዱ እንደሆኑ ምን ያህል ጊዜ ተሰምቶልዎት ያውቃል? | 0 1 2 3 4 |  |
| 6 | ባለፈው ወር ውስጥ ማድረግ ያለብዎትን ነገሮች ሁሉ መቋቋም (መቆጣጠር) እንደማይችሉ ምን ያህል ጊዜ አግኝተዋል? | 0 1 2 3 4 |  |
| 7 | ባለፈው ወር ውስጥ ምን ያህል ጊዜ በሕይወትዎ ብስጭትን መቆጣጠር ቻሉ? | 0 1 2 3 4 |  |
| 8 | ባለፈው ወር ውስጥ እርስዎ በነገሮች ላይ የበላይ መሆንዎን ምን ያህል ጊዜ ተሰማዎት? | 0 1 2 3 4 |  |
| 9 | ባለፈው ወር ውስጥ ከቁጥጥርዎ ውጭ ከሆኑ ነገሮች የተነሳ ምን ያህል ጊዜ ተቆጥተዋል? | 0 1 2 3 4 |  |
| 10 | ባለፈው ወር ውስጥ ችግሮች ለማሸነፍ እስከማይችሉ ድረስ በጣም ከፍተኛ ሆነው እየመጡ እንደሆነ ምን ያህል ጊዜ ተሰማዎት? | 0 1 2 3 4 |  |
|  | **ክፍል IV: ልኬቶች** |  |  |
| 401 | ክብደት _________ ኪ.ግ. |  |  |
| 402 | ቁመት _________ ሴሜ |  |  |
| 403 | የወገብ ስፋት _________ ሴሜ |  |  |
| 404 | የዳሌ ዙሪያ _________ ሴሜ |  |  |
| 405 | ሴረም ትራይግላይሰራይድ _________ mg / dl |  |  |
| 406 | ሴረም ኮሌስትሮል _________ mg / dl |  |  |
| 407 | ሴረም ኤች ዲ ኤል _________ mg / dl |  |  |
| 408 | ሴረም LDL _________ mg / dl |  |  |
| 409 | ክሬቲናይን ______ |  |  |
| 410 | FBS 1______ FBS 2______ FBS 3______ FBS4______ |  |  |
| 411 | HA1C ______ |  |  |
| 412 | የደም ግፊት መጠን ከመዝገብ | ስይስቶሊክ________  ዳያስቶሊክ________ |  |
| 413 | ንባብ 1 | ስይስቶሊክ________  ዳያስቶሊክ________ |  |
| 414 | ለደም ግፊት መድኃንት ይወስዳሉ | 1. አዎን  0. አይደለም |  |
| 415 | የደም ግፊት ህመም ሁኔታ | 1. አለ 🡪516  0. የለም 🡪 አመሰግናለሁ ጨርሰናል |  |
| 416 | ለደም ግፊት ታማሚዎች ብቻ፡ የደም ግፊት እንዳለብዎት ካወቁ ወዲህ ምን ያክል ጊዜ ሆነ? | ­­____ዓመት |  |

- ***ከመዝገብ የሚወሰዱ መረጃዎች***
