# Supplementary material for: Determinants of hypertension among patients with type 2 diabetes mellitus on follow-up at Tikur Anbessa Specialized Hospital, Addis Ababa: A case-control study
Source: PLoS One. 2021 Aug 23;16(8):e0256399. doi: 10.1371/journal.pone.0256399 (PMC8382175; doi:10.1371/journal.pone.0256399)
Supplement: Attachment — Submitted filename: Rebuttal Letter2.docx [file pone.0256399.s004.docx]

Dear reviewers and editor,

Thank you for your review and important issues you raised in the paper.

We have read the reviewer's comments carefully and hope that the revised version now submitted will be regarded as having enhanced the previous version. The authors very welcome the reviewer's comments and suggestions. These contributions have appreciably improved the final paper quality. Hereunder, kindly get the responses to the points raised under each point.

**Response to reviewer #1:**

Review Comments to the Author

Reviewer #1: In table 2, units of mean SBP and DBP were mm Hg. This units need no space between mm and Hg. 'mmHg'

Comment: the spaces were removed and corrected as mmHG based on the reviewer’s comment.

In table 3, AOR in DM duration was 1.035(1.01, 1.07). AOR was needed to unified two alignment.

Comment: the decimal place was rounded to two places based on the reviewer’s comment.

**Response to Reviewer #2:**

Reviewer #2: Thank you again for presenting the revised form of your work. The authors have revised the manuscript incorporating comments suggested. I advise the inclusion of 'materials' in the methods sub-section of the abstract. Also please check the acknowledgement section for patients, while offering their full consent and participation, should have been acknowledged, or if that has been addressed elsewhere.

Comment: materials was now included in the abstract subsection methods, and the study participants were also acknowledged in the revised version based on the reviewer’s comment.

Thank you for your consideration. We look forward to hearing from you.

Sincerely,

The corresponding author
